# Social Capital, Technological Empowerment, and Resilience in Rural China

**DOI:** 10.3390/ijerph182211883

**Published:** 2021-11-12

**Authors:** Chao Wang, Tao Zhang, Wendong Xu, Haibo Ruan, Jiayi Tang

**Affiliations:** 1 School of Public Policy & Management (School of Emergency Management), China University of Mining and Technology, Xuzhou 221116, China; wangchaoccnu@163.com; 2 School of Public Administration and Human Geography, Hunan University of Technology and Business, Changsha 410205, China; zhangtao8912@hotmail.com; 3 School of Foreign Studies, China University of Mining and Technology, Xuzhou 221116, China; xwdspace@163.com; 4 Institute of China Rural Studies, Central China Normal University, Wuhan 430079, China; 5 School of International Relations & Public Affairs, Fudan University, Shanghai 200433, China

**Keywords:** social capital, technological empowerment, pandemic resilience, social trust

## Abstract

In the post-pandemic era, the need for resilient and flexible COVID-19 prevention strategies in rural areas has become increasingly prominent. Based on a sample of 2229 rural residents nationwide, the Structural Equation Model was adopted to analyze the influence of social capital and technological empowerment on pandemic resilience in rural areas. The proportion of diversity, adequacy, and effectiveness of pandemic prevention measures taken by communities was about 57%. Social capital (0.667) and technological empowerment (0.325) had a significant positive impact on rural resilience and pandemic prevention. Social capital plays a mediating role between technological empowerment and pandemic resilience in rural areas. The risk of disease in society stimulates the inherent social capital factors in villages, with the individual social network generating strong social support. Technological empowerment can not only provide new methods for the connection of social capital, but also bring new means for rural authorities to improve their governance capabilities. Social trust in social capital plays an important role in rural resilience and pandemic prevention. The indirect effect of technological empowerment through social capital on pandemic resilience is greater than its direct effect. Social capital construction is the key to rural resilience and pandemic prevention.

## 1. Introduction

COVID-19 is a once-in-a-century health crisis that will affect people for decades to come. The wild spread of the virus has posed a huge challenge to China’s grassroots governance system and pandemic prevention system. During the outbreak in Wuhan, about 5 million people left Wuhan to go back home, the vast majority of which returned to rural China [1]. Rural pandemic prevention is regarded as a crucial influence on overall pandemic prevention nationwide. By the end of 2020, there were 509.79 million rural residents in China, distributed in 690,000 administrative villages. However, there were only about 610,000 village-level clinics, showing the unfair distribution of public health facilities in rural areas (see Table 1). Therefore, many scholars have stressed the importance of issues in rural areas when dealing with the pandemic, such as backward medical and health facilities [2], poor medical and health conditions [3], lack of prevention and control materials, and villagers’ lack of awareness of prevention and control [4].

Carl Folke and others emphasized that, in the context of a health resource shortages, resilience is particularly urgent during events such as the COVID-19 pandemic, and it is necessary to build up community resilience [5]. Resilience in rural areas is increasingly being valued by government and society [6], which begs the question: what is rural resilience and what are the influencing factors? The concept of resilience comes from physics and refers to an object’s elasticity and resistance to stress. According to S.B. Manyena, resilience used to refer to the needs and vulnerabilities in a community, but now it tends to reference the recovering ability of a community after a disaster, especially in the case of external assistance [7]. L. Butler also emphasized resilience—the ability to adapt, recover, and to return to a normal track after a crisis [8].

So far, resilience, integrated with rural construction and pandemic prevention and control, has become part of grassroots governance and risk prevention and control. Based on the above analysis of the concept and characteristics of resilience, the concept of “pandemic resilience” was proposed: rural areas can constantly develop pandemic prevention and control measures according to dynamic situations, fully link internal and external resources, give full play to the strength of rural residents, meet the different needs of pandemic prevention and control, provide livelihood support and economic and social development in rural areas, and quickly recover and achieve all-round development.

Sonny S. Patel et al. identified nine core elements of community resilience: local knowledge, community networks and relationships, communication, health, governance and leadership, resources, economic investment, preparedness, and spiritual outlook [9]. Bian Yanjie analyzed the impact of social capital on pandemic prevention in rural areas. Based on the concept of antiviral social capital, Bian divided these elements by their intensity and breadth of social connections. Those with higher antiviral social capital performed better in pandemic prevention and control [10]. Kokubun K. and Yamakawa Y.’s research shows that social distancing is not enough to control the spread of the pandemic, and social capital makes up for it [11]. China’s rural areas are typical acquaintance societies. Therefore, it is necessary to continue studying the impact of social capital on resilience in rural areas.

The Chinese government implemented a plan, the Outline of the Strategy for Digital Rural Development in 2019, to accelerate the development of broadband communication networks, mobile internet accessibility, digital television networks, and next-generation internet in rural areas. At present, the value of rural digitalization construction has found its way into COVID-19 prevention and control. For example, information technology plays an important role in identifying infection sources, predicting disease spread, and remote diagnosis. As a result, the era of public health emergency prevention and control, led by information technology, has arrived [12]. Some researchers have analyzed the application of Health Code technology. By tracking the trajectory of population flow through Health Code, technology companies can monitor population control, improve the government’s grassroots governance ability, and realize technological empowerment [13]. However, the role of digital rural construction in rural pandemic prevention and control has not received sufficient attention in theoretical research. Therefore, this research intends to establish a model to explore the influence of social capital and technological empowerment on pandemic resilience in rural areas, and to discuss the mediating effect of social capital between technological empowerment and pandemic resilience.

## 2. Theoretical Background and Hypothesis Development

### 2.1. Pandemic Resilience in Rural Areas

The resilience of pandemic prevention in rural areas describes the comprehensive resilience, recovery, and development of rural areas in pandemic prevention and control, economic development, and livelihood protection. The original rural pandemic prevention and control only followed a single index, which only focused on pandemic prevention and control, as well as villagers’ health as the first value choice. However, other needs of rural residents cannot be met, such as their demands in living, psychological, and occupational aspects. Rural areas have come to a standstill due to pandemic prevention and control and have even experienced regression. While improving the level of pandemic prevention in rural areas, it is also necessary to focus on building resilience capacity in rural areas to combine dynamic pandemic prevention with sustainable development.

### 2.2. Social Capital

As Defined by Robert D. Putnam, social capital “refers to the characteristics of social organization, such as trust, norms, and networks, which enhance social efficiency by promoting coordination and action. Social capital increases the return on investment in physical and human capital.” [14] Social capital forms a network of relationships between social members and forms reciprocal relationships, which are embodied in the acquaintance society and human society in traditional Chinese villages. The accumulation of social capital among village members can promote them to participate in grassroots governance and improve the efficiency of pandemic prevention and control [15]. Some scholars believe that rural residents’ social capital can be divided into three dimensions: social network, social trust, and social participation [16].

Social network refers to the relationship-type society formed among rural households, such as relatives, countrymen, friends, mentoring, and so on. Individuals can form different relationship networks through blood relationships, kinships, occupations, interests, and other channels. The richer the relationship network, the wider the social scope. At the same time, social networking is an important carrier of social capital, and the realization of the value of social capital needs to be based on social networks. Social members can establish a sense of identity and belonging through the relationship network, and then enhance social responsibility because of member interaction. The close social relationship between villagers can broaden access to information and reduce the cost of information dissemination. Research conducted by Melinda Moore et al., based on 10 countries, shows that social networks are of great significance for building resilience in communities [17].

Social trust is the interpersonal trust relationship formed in a relational society, and it is the trust cognition formed by farmers in long-term continuous communications. The social trust of rural residents is reflected in two aspects: one is the social trust among residents, e.g., trust can reduce farmers’ perception of risks in the future [18] and promote voluntary activities; the other is trust between residents, village cadres, and grassroots organizations. Social trust can also serve as an informal system, playing the role of “lubricant” in interpersonal communication. Trust can shorten the distance between people, and expand the radius of individual trust [19]. Emotional trust, relational trust, and institutional trust are three trust structures in Chinese society. Compared with institutional trust in urban stranger society, trust in rural acquaintance society is an emotional and relationship trust based on consanguinity, relatives, and friends, which have traditional factors and historical and cultural factors. It is generally believed that interpersonal trust is high in rural areas, and a high level of social trust can promote the implementation of pandemic prevention and control policies.

Social participation refers to the participation of farmers in rural public affairs. Rural households coexist in a shared rural community with the same identity cognition, values, and emotional concepts, and they share weal and woe with each other [20]. Kathryn M. Barker et al. found that active social participation of residents improved relations with health authorities and increased trust in the health system’s pandemic prevention efforts, thus better coping with public health crises [21]. Eve Coles and Philip Buckle pointed out that a community’s resilience and recovering ability have multidimensional attributes. Only those people affected by disasters have the ability, knowledge, skills, and channels to participate, making their participation meaningful and effective in building community resilience [22]. In the face of the pandemic, villagers’ active participation in pandemic prevention and control can increase the pandemic prevention capacity of villages and accumulate social capital in the process of participation.

In China, rural social capital has rich historical roots in dealing with and responding to public disasters. Villages are living communities based on blood relationship and geography. Members help each other and can save each other in the face of public security crises. “In the long history of China’s smallholder farmers, a complete set of social assistance systems has been formed to support the survival and continuation of smallholder farmers” [23], such as blood assistance, squire assistance, and villagers’ mutual assistance. In the face of the pandemic, social capital is an important mechanism for improving resilience in rural areas. For individuals, social networks and social capital play a positive role in obtaining pandemic information, maintaining mental health, and obtaining social support [24]. For rural pandemic prevention, the more abundant social capital is, the more farmers can actively participate in pandemic prevention, control work, and increase the confidence of overcoming the pandemic. Based on this, the first hypothesis of this study is proposed:

**Hypothesis** **1** **(H1).** *There is a positive and significant relationship between social capital and pandemic resilience in rural areas.*

### 2.3. Technological Empowerment

In 2021, the Chinese government proposed to “implement the digital rural construction and development project” and “strengthen the digital and intelligent construction of rural public services and social governance.” Digital rural construction will introduce new technologies such as big data, cloud computing, block chain, artificial intelligence, and 5G to rural areas and bring technology to rural social governance. With the development of a modern market economy, rural households are individualized and atomized and act in accordance with rational principles. The logic followed by technology governance is precision and refinement, including accurate identification and scientific analysis of problems to help administrators improve rural governance capacity, that is, to give technology to administrators [25].

In pandemic prevention and control, the function and role of big data and technology has shown its influence. First, technology enhances the ability to accurately identify villages. Through big data, Internet, and 5G technology, the development and spread of the virus can be accurately traced, tracked, located, and recorded; the individual trajectory can be realized with technology, so as to achieve accurate identification of infected cases, accurate isolation, prevention, and control. In essence, the monitoring capacity of rural administrators on the pandemic situation would improve. Second, technology improves analytical capabilities. In the face of complicated information of public opinion, news reports, and the pandemic situation, big data technology clarifies and classifies information through its unique algorithm model, identifies the health information status of people in certain regions according to pandemic statistics [24], and divides them into different color codes, such as red code, yellow code, and green code. This technology greatly improves the efficiency and utilization of information processing to achieve dynamic management and strengthen the identification of risk areas. Finally, technology enhances rural governance capacity. Because of technology, recognition ability and analytical ability have greatly enhanced the governance capability of village authorities. For instance, in the post-pandemic era, big data technology can help village authorities obtain the demands of rural residents in a timely manner to quickly and accurately allocate and mobilize various materials, thereby improving the effectiveness and efficiency of governance. Therefore, technology landing in rural areas and digital rural construction play a positive role in pandemic prevention and control, as well as rural resilience. Based on this, the second hypothesis is proposed:

**Hypothesis** **2** **(H2).** *There is a significant positive correlation between technological empowerment and pandemic resilience in rural areas.*

### 2.4. Technological Empowerment and Social Capital

The relationship between information technology and social capital presents two different views. The first view is that the use of personal information technology will inhibit the formation and development of social capital. Because the construction of traditional social capital is based on consanguinity, geography, and industry, its construction form is mostly face-to-face contact, communication, and interaction. However, the development of information technology has increased isolating activities during personal time, such as reading newspapers and watching TV, which has reduced face-to-face communication with relatives and friends [26]. Individuals who use the Internet for a long time will reduce their enthusiasm for participating in social communication, resulting in psychological loneliness, fear, and other social emotions [27].

The second view is that information technology will increase individual social capital, and information technology will play a positive role in promoting it. The use of network technology will break the traditional form of face-to-face communication and provide a new virtual space for members to communicate on the network platform, forming flatter interpersonal relationships [28]. Katz et al. found that anonymity of internet use can improve the participation and trust of internet users [29]. Jeni Warburton, in her research on the use of information technology by the elderly, found that the increase in the use of mobile phones and the Internet will bring more emotional support to the elderly. Based on this research, information technology increases the social capital of the elderly [30]. At the same time, the network can transcend time and space limitations of communication and interaction, increase the closeness of connection, and reduce the cost of social capital formation. When using the information technology platform, users can selectively use it according to their own interests and hobbies, and join the corresponding network organization. From the level of social organization, it is also conducive to the formation of social capital.

In order to reduce the occurrence of clustering events and to block the transmission channels of the virus, face-to-face social communication is limited to a certain extent, but communication through information technology can help block transmission channels. Therefore, in the pandemic, the use of information technology can promote the accumulation of social capital, which has a positive and significant effect. Based on this, the third hypothesis of this study is proposed:

**Hypothesis** **3** **(H3).** *Social capital plays an intermediary effect between technological empowerment and pandemic resilience in rural areas.*

**Figure 1:**
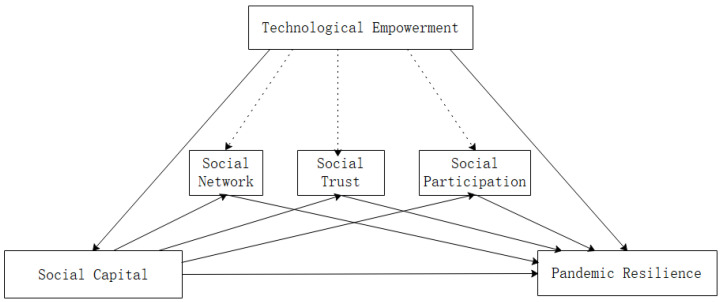
Conceptual model.

Figure 1 presents the conceptual model of the entire research hypothesis.

## 3. Methods

### 3.1. Data Sampling

Stratified sampling and random sampling were used in this study. First, one city (i.e., 28 provinces × 1 city) was randomly selected from each province for investigation. Second, 10 rural communities were randomly selected in each city (i.e., 28 provinces × 1 city × 10 rural communities). Finally, a total of 80 community residents and community-related workers were randomly selected from the selected rural communities (i.e., 28 provinces × 1 city × 10 rural communities × 8 respondents). Due to the limitations of pandemic prevention and control, online surveys are the main method of data collection, supplemented by offline surveys. Considering the relative uncertainty of online questionnaire surveys, the research team randomly selected 10% of respondents for a return visit to test the quality of questionnaire data to ensure the reliability of the survey data. A total of 2240 questionnaires were distributed in the survey. After removing 11 invalid questionnaires manually, 2229 valid questionnaires were finally collected, with an effective rate of 99.5%, reaching the requirement of 90%.

Among these 2229 respondents (see Table 2), male respondents accounted for 47.60% and female respondents accounted for 52.40%. In terms of age, those aged from 18 to 25 accounted for 24.45%, those aged from 26 to 40 accounted for 13.77%, those aged from 41 to 60 accounted for 31.94%, and those over 60 accounted for 26.83%. The respondents are mainly young and middle-aged. In terms of occupations, farmers and workers accounted for 54.02% and 34.28% respectively. In terms of marital status, unmarried respondents accounted for 30.82%, married respondents accounted for 66.76%, and the rest were divorced or widowed. Generally speaking, the sample of 2229 respondents meets the relevant requirements of statistics and can be analyzed statistically.

### 3.2. Measurements

In this study, the impact of social capital and technological empowerment on pandemic resilience in rural areas was investigated by taking rural flexible pandemic prevention measures as endogenous latent variables and taking social capital and technological empowerment as exogenous latent variables. Meanwhile, social capital is used as the intermediary variable between technological empowerment and pandemic resilience in rural areas, and the indirect effect of technological empowerment on pandemic resilience through social capital is investigated. Social capital is divided into three dimensions, and the effect of the three dimensions is explored. Specific settings are as follows:

#### 3.2.1. Pandemic Resilience

The questionnaire examines pandemic resilience in rural areas according to ten aspects, as shown in Table 3, mainly focusing on three aspects: one is the situation of rural pandemic prevention and control, community involvement in pandemic prevention work, household health screenings for villagers, community health monitoring, full comprehension of the pandemic period potential risk, and cadres holding leadership positions; second, information on pandemic prevention and control measures, including their diversity, adequacy, and effectiveness; and third, paying attention to the livelihood needs of villagers, such as understanding their living difficulties during the pandemic and responding to their living needs. In terms of the answers to the questions, a five-level Likert scale was used, which was successively set as “Very poor = 1; Poor = 2; General = 3; Good = 4; Very good = 5”. The higher the response score, the better the resilience of the community.

#### 3.2.2. Social Capital

As introduced in the theoretical basis of this paper, social capital is divided into three dimensions, namely: social network, social trust, and social participation; each dimension is measured by three aspects. There is a hierarchical progressive relationship between the three dimensions. People with rich social networks will bring higher social trust, and then promote their social participation, whether formal or informal. The social network is mainly investigated from the communication between villagers and relatives, village cadres, and friends. Social trust was measured by examining villagers’ trust in relatives, village cadres, and the community. Social participation mainly examines the situation of residents’ participation in community pandemic prevention work, as well as the clear relationship between each participant and the division of responsibilities of each participant. A five-level Likert scale was used to record answers, which were successively set as “Very poor = 1; Poor = 2; General = 3; Good = 4; Very good = 5”. The higher the score, the better the social capital situation.

#### 3.2.3. Technological Empowerment

The questionnaire measures technological empowerment from four aspects, including the community’s adoption of modern technology in pandemic prevention and control, the establishment of a villager physical information database, community network communication, and community WeChat group communication. A five-level Likert scale was used to record answers, which were successively set as “Very poor = 1; Poor = 2; General = 3; Good = 4; Very good = 5”. The higher the score, the better the social capital situation.

### 3.3. Analytical Methods

This study was conducted by adopting descriptive statistics, a Structural Equation Model (SEM), and mediation effects. First, the overall situation of pandemic resilience in rural areas was examined through descriptive statistical analysis, and different evaluations of specific pandemic prevention measures were compared. Second, SPSS24.0 (IBM, Armonk, NY, U.S.A.) was used to test the reliability of each scale, and Amos 24.0 (IBM, Armonk, NY, U.S.A.) was used to construct a Structural Equation Model with pandemic resilience as the endogenous latent variable and social capital and technology as the exogenous latent variables. Parameters were estimated using the maximum likelihood method. The model evaluation indexes included the chi-square degree of freedom ratio (CMIN/DF), the mean square of residual and square root index (RMR), the mean square of progressive residual and square root index (RMSEA), the value-added fit index, and reduced the fit index. Finally, the hypothesis was tested by analyzing the fitting index and path coefficient of the model.

## 4. Results

### 4.1. Statistical Analysis of Rural Resilience and Pandemic Prevention

As shown in Table 4, among the 10 observational variables, the proportion of communities monitoring the physical condition of key groups (R3) at the “very-good level” is the highest, reaching 62.85%; the second is communities taking the initiative to carry out community pandemic prevention work (R1) and communities’ leading cadres sticking to their posts and leading from the front (R7), both of which are “very good”, accounting for 60.03%. However, the proportion of respondents that felt there was a community initiative to understand the villagers’ living difficulties (R4) during the pandemic was only 53.75%, which was the lowest among all the observed variables. At the same time, the proportion of respondents that felt the community pandemic prevention work responding to villagers’ living needs (R5) and the community fully knowing the potential risks during the pandemic period (R6) was only 54.96%, just higher than R4. The percentages of diversity (R8), adequacy (R9), and effectiveness (R10) of pandemic prevention measures taken by communities were 57%, which was not very high.

### 4.2. Test of Models

**Figure 2:**
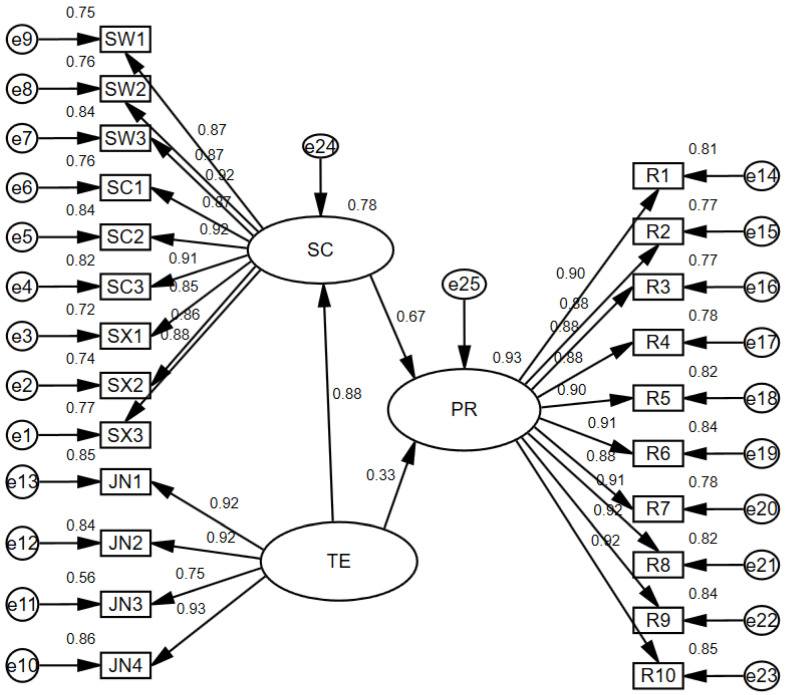
Structural model for PR. PR, Pandemic resilience; SC, Social Capital; TE, Technological of Empowerment.

Qualified reliability of the questionnaire scale is the premise of statistical analysis, and high reliability guarantees reliable analysis. Therefore, the reliability of each subscale was detected by SPSS24.0, and the data results showed that Cronbach’s Alpha coefficients of pandemic resilience for social capital and technological empowerment subscales were 0.976, 0.969, and 0.929, respectively. The reliability coefficients of the three subscales are all above 0.9, indicating that the subscales have high reliability. To explore the relationship between social capital and technological empowerment and the resilience of measures taken in rural pandemic prevention, AMOS24.0 software was used to establish a Structural Equation Model of the resilience of pandemic prevention in rural areas, as shown in Figure 2.

Table 5 shows the fitting of the Structural Equation Model of the flexibility of pandemic prevention and control in rural areas. The chi-square degree of freedom ratio of the model is 11.965 (greater than 3) because this value is greatly affected by the respondent size and is still within an acceptable range. The RMR value and RMSEA value of the model meet the requirements, which are 0.013 and 0.070, respectively. Combined with the fitting results of the value-added fit index and the reduced fit index of the model, it can be considered that the overall fit of the model is good, and the path analysis can be carried out.

Figure 2 shows the standardized coefficients of each path. Between the observed variables and the latent variables, only one factor load coefficient is less than 0.8, and the rest are above 0.8, indicating that the observed variables can reliably represent the latent variables. Table 6 reports the specific path coefficients and standard errors. The C.R. values of all observed variables are greater than 1.96, and the path coefficients between latent variables are all positive and significant, indicating the existence of a positive influence.

Social capital has a remarkably positive influence on pandemic resilience, with an effect value of 0.667, confirming hypothesis H1. With the improvement of villagers’ social capital, the level of pandemic prevention in rural areas will gradually improve. The village is originally a society of acquaintances, and the villagers are closely connected and live in a community. In the face of the spread of the pandemic, the existing idle social capital stock is stimulated, and the villagers actively participate in pandemic prevention and public services in rural areas, which is conducive to the prevention and control of the pandemic and the restoration of rural order after the pandemic. At the same time, the social relationship of acquaintances can also repair the social psychology traumatized by the pandemic and play a role of emotional comfort. In addition, as an endogenous driving force, social capital has a strong ability to adapt and recover, and can link, mobilize, and integrate resources in a certain region, which plays an important role in rural pandemic prevention and control, in addition to being a feature of the resilience of pandemic prevention in rural areas.

Technological empowerment has a significant positive effect on pandemic resilience, with an effect value of 0.325, proving hypothesis H2. Modern technology has changed the tools and means of rural administrators, turning fuzzy identification into precise identification, and improving the efficiency of pandemic prevention and control and reducing the cost of control [31]. At the same time, the application of big data technology in rural areas can help strengthen their communication with the outside world, obtain the latest information, and acquire advanced knowledge and skills of pandemic prevention. Through online communication, the modern network has greatly reduced the risk of spreading the pandemic and has contributed to pandemic prevention in rural areas. Information technology has been used to realize the perception and intelligent processing of pandemic information, improve the early warning capacity of villages, and contribute to the resilience of pandemic prevention.

### 4.3. Test of Mediating Effects

In order to explore the intermediary effect of social capital between technological empowerment and pandemic resilience, the bootstrap function of AMOS 24.0 software is used as a test. The number of bootstrap samples is set to 10,000 and the confidence interval is set to 95%. According to the suggestions of Preacher and Hayes [32], the upper and lower confidence intervals are calculated to test whether the indirect effect is significant. Table 7 reports the test results of the bootstrap function. First, technological empowerment has a significant impact on pandemic resilience in rural areas, and its direct effect value is 0.325; second, technological empowerment has an indirect impact on pandemic resilience through social capital, and the effect value is 0.590; finally, the total effect value of technological empowerment on pandemic resilience is 0.915, and the two-sided test results of direct impact, indirect influence, and overall influence are significant. The above three aspects meet the conditions of an intermediary effect test, which shows that social capital plays an intermediary effect between technological empowerment and pandemic resilience and, consequently, proves hypothesis H3.

Finally, the results of hypothesis testing are shown in Table 8, and H1, H2, and H3 are verified.

## 5. Discussion

To explore the impact of social capital and technological empowerment on pandemic resilience in rural areas, this study uses the Structural Equation Model as an analysis tool to explore the relationship between the three. The model results show that social capital and technological empowerment have a significant positive impact on pandemic resilience. Social capital plays an intermediary effect between technological empowerment and pandemic resilience. These facts prove the hypotheses H1, H2, and H3 proposed in this article. Social capital under the pandemic can bring external support to individuals, including material and spiritual support. These supports increase the resilience of rural pandemic prevention. Technological empowerment exists as a governance tool and improves rural governance capabilities.

Research hypothesis H1 confirms that the three aspects of social capital—social network, social trust, and social participation—have a positive and significant impact on pandemic resilience in rural areas, which is the same as the results of Kokubun K. and Yamakawa Y. [11]. In terms of the factor load of social capital, listed in descending order, the load is social trust, social participation, and social network. It can be concluded that social trust plays a major role in affecting resilience and pandemic prevention. The virus has led to indifference and isolation among members of society, which creates distrust. In addition, social trust, including the villagers’ trust in their relatives, trust in rural cadres, and trust in the grassroots government [33], is the key to victory over the pandemic. Social trust can build a sense of moral obligation among villagers, increasing attention paid to others [34] and stimulate villagers to actively participate in pandemic prevention and control [35]. A social network that includes a wide range of individuals can obtain more social support during the pandemic, such as material assistance and psychological comfort. Social trust can be internally transformed into individual confidence in overcoming the pandemic and hope for a better life in the future. The pandemic crisis has stimulated the improvement of individuals’ public minds and sense of responsibility. More villagers have participated in the prevention and control of the pandemic, and as a result, rural community awareness has increased.

Research hypothesis H2 confirms that technological empowerment has a significant positive impact on pandemic resilience in rural areas. Zhiyao Wang and others believe that the use of modern technology to prevent major risks such as pandemics can improve risk prevention and control capabilities at the governance level [36]. This article agrees with these authors’ point of view that modern information technology can be embedded in rural society as a means of governance, which improves the governors’ ability in information collection and information transmission, and improves the efficiency of the entire governance process. Based on the advantage of big data and integrating the collected information, modern information technology can realize dynamic monitoring and accurately identify risk areas and close contacts. These applications have greatly improved the efficiency of pandemic prevention and control.

Research hypothesis H3 confirms that social capital plays an intermediary role between technological empowerment and pandemic resilience in rural areas. The establishment of traditional rural social capital is based on face-to-face communication and interaction, such as labor exchange and assistance in neighboring agricultural production, gatherings in household life, and celebrations held on festivals. However, these forms of offline communication were blocked by the pandemic. Modern information technology provides new forms and channels for communication between members in a state of pandemic isolation, which can realize information resource sharing and visualization. These exchanges have a positive effect on the construction of social capital among members [37]. The online communication form provided by modern information technology provides a new way for villagers to contact each other, which happens to have a progressive effect. The communication and mutual care of the villagers through the Internet have consolidated their relationship and promoted the construction of the villagers’ resilience psychology [38]. At the same time, modern information technology creates an online communication platform for villagers to meet the emotional needs of other villagers, providing comfort to the villagers and support to the society [39].

It is worth noting that the direct effect of technological empowerment on pandemic resilience is smaller than the indirect effect. This may be related to the characteristics of modern information technology. Modern information technology, for the village, is a tool or resource embedded exogenously. However, social capital is an endogenous resource in the village. Therefore, the social capital in the village itself has unique advantages. The relationship of acquaintances in Chinese rural society gives birth to a unique culture of social capital. The interweaving and superposition of blood relationships, geographical relationships, kinships, etc., unlocks the potential for social capital development. In a normal society, the communication between villagers is relatively close; there are frequent contacts, and the factors of social capital are already being shaped. However, the arrival of an abnormal society has stimulated the underlying factors of social capital. The villagers have more trust and help each other to weather difficulties together. Consequently, the scenario of traditional villages jointly resisting risks can be reproduced. Modern information technology provides tools and means for stimulating social capital. It not only provides rural authorities with methods for pandemic prevention and control, but also improves the ability of rural authorities to respond to pandemic risks.

## 6. Limitations

There are three limitations in this study. First, the data used in this study was collected using a one-round survey, and survey data is not continuously tracked, so the data can only reflect the current situation. Second, the variables used in this study may also be affected by other factors, which requires further exploration. Finally, there are many factors influencing pandemic prevention resilience in rural areas. This study only explored two variables, and there are other aspects worth exploring.

## 7. Conclusions

This research is based on a survey of 2229 community residents and community-related workers in rural China, using the Structural Equation Model as an analysis tool to explore the impact of social capital and technological empowerment on pandemic resilience in rural areas. The research has shown that social capital and technological empowerment have a significant positive impact on pandemic resilience. Social capital plays an intermediary effect between technological empowerment and pandemic resilience. Hypotheses H1, H2, and H3 have all been confirmed. The social crisis brought about by the pandemic has activated social capital factors stored in the society of acquaintances. Individual social networks provide social support for overcoming the pandemic, which is conducive to overcoming the vulnerability of individuals and increasing the resilience of villages in pandemic prevention through social relations. Modern information technology has become an important builder of social capital in the pandemic. It plays an important role in reducing information asymmetry, transmitting pandemic information, and popularizing pandemic prevention knowledge. At the same time, technical empowerment helps rural authorities achieve refined governance, traceability throughout process, and precise identification. All these activities have strengthened the governance ability of the governance subjects and have empowered rural authorities. Therefore, to build a pandemic resilience system in rural areas, rural authorities must not only explore the potential of traditional rural acquaintances’ social capital, but also promote the construction of rural information technology systems and cultivate the ability of rural cadres to use modern information technology flexibly. At the same time, it is equally meaningful to organize both online and offline cultural and festival activities during the pandemic, so as to promote communication and interaction between rural residents, thus establishing a shared emotional community in rural areas.

## Figures and Tables

**Table 1:** Village health settings.

	Year (year)	2016	2017	2018	2019
Index (unit)					
Number of village clinics		638,763	632,057	622,001	616,094
Number of village clinics run by villages		351,016	349,025	342,062	339,525
Number of village clinics set up by township hospitals		60,419	63,598	65,495	69,091
Number of village clinics run jointly		29,336	28,687	28,353	27,626
Number of villages with health clinics in administrative villages (%)		92.9	92.8	94.0	94.8

Data source: Official website of the National Bureau of Statistics.

**Table 2:** Characteristics of respondents.

Characteristics of the Indicators	Classification	Frequency	Proportion (%)	The Standard Deviation
Gender	Male	1061	47.60	0.50
	Female	1168	52.40	
Age	Under the age of 18	67	3.01	1.51
	18~25 years old	545	24.45	
	26~40 years old	307	13.77	
	41~60 years old	712	31.94	
	More than 60 years of age	598	26.83	
Identity and occupation	Farmers	1204	54.02	0.76
	Workers	764	34.28	
	Private business owner	211	9.47	
	Village authorities	43	1.93	
	Other	7	0.30	
Marital status	Single	687	30.82	0.52
	Married	1488	66.76	
	Divorced	44	1.97	
	Widowed	10	0.45	
Total		2229	100	

**Table 3:** Construct measurement.

Construct	Item	Coding	Measurement
Pandemic resilience	Community initiatives and timely implementation of community pandemic prevention	R1	Very poor = 1; Poor = 2; General = 3; Good = 4; Very good = 5
	The situation of community to enter villagers’ house and check their health conditions	R2	
	Community monitoring of health conditions of key groups	R3	
	The community took the initiative to learn about the villagers’ living difficulties during the pandemic	R4	
	Community pandemic prevention work responds to the living needs of villagers	R5	
	Communities are fully informed about potential risks during the pandemic	R6	
	Community leading cadres stick to their posts and command from the front	R7	
	Diversity of community immunization measures	R8	
	Adequacy of community preventive measures	R9	
	Effectiveness of community vaccination measures	R10	
Social capital	Communication between villagers and relatives	SW1	
	Communication between villagers and village cadres	SW2	
	Communication between villagers and various friends	SW3	
	Trust in relatives	SX1	
	Community residents trust the village cadres	SX2	
	Community trust in the community during the pandemic	SX3	
	Residents’ participation in community pandemic prevention	SC1	
	Clear information on the relationship between various participants in community pandemic prevention work	SC2	
	Division of responsibilities among participants in community pandemic prevention work	SC3	
Technological empowerment	Application of modern information technology in community pandemic prevention and control	JN1	
	Community establishment of villagers’ physical condition information database	JN2	
	Community network communication	JN3	
	Community WeChat group communication	JN4	

**Table 4:** Descriptive analysis of pandemic resilience, N (%).

Pandemic Resilience	Very Poor	Poor	General	Good	Very Good
R1	0.49	2.33	8.93	28.22	60.03
R2	0.85	3.36	11.75	27.23	56.81
R3	0.45	3.23	7.76	25.71	62.85
R4	0.67	3.77	14.13	27.68	53.75
R5	0.54	3.41	12.07	29.03	54.95
R6	0.72	3.32	11.93	29.07	54.96
R7	0.67	2.33	9.56	27.41	60.03
R8	0.63	2.87	11.62	27.59	57.29
R9	0.54	2.69	10.50	29.03	57.24
R10	0.45	2.42	10.45	28.89	57.79
Respondent	2229				

**Table 5:** Summary of the fit indices.

The Evaluation Index	Optimal Standard Value	Model Values	Results
Absolute fit index			
Chi-square value (CMIN)		2716.160	
Chi-square degree of freedom ratio (CMIN/DF)	Greater than 1 and less than 3	11.965	fair
Residual mean square and square root exponent RMR	<0.05	0.013	ideal
Progressive residual mean square and square root RMSEA	<0.08	0.070	ideal
Goodness of fit index GFI	>0.9	0.896	fair
Adjust the goodness of fit AGFI	>0.9	0.873	fair
Value-added fit index			
Standard fit index NFI	>0.9	0.961	ideal
Relative fit index RFI	>0.9	0.957	ideal
Incremental fit index IFI	>0.9	0.964	ideal
Nonstandard adaptation index TLI	>0.9	0.960	ideal
Comparison of fit index CFI	>0.9	0.964	ideal
Reduced fit index			
Pared-down fit index PGFI	>0.5	0.737	ideal
Simplified adjustment of the regulation alignment index PNFI	>0.5	0.862	ideal
Pared-down comparison fitting index PCFI	>0.5	0.865	ideal
CN values	>200	216	ideal

**Table 6:** Path coefficients for the hypothetic model towards PR.

Path Constructs			SF	NSC	S.E.	C.R.	*p*	Assuming That
SW1	<--	Social capital	0.878	1.000			***	
SW2	<--	Social capital	0.862	1.039	0.018	58.093	***	
SW3	<--	Social capital	0.846	0.926	0.017	55.885	***	
SX1	<--	Social capital	0.906	1.065	0.016	64.849	***	
SX2	<--	Social capital	0.916	1.079	0.016	66.628	***	
SX3	<--	Social capital	0.870	1.029	0.017	59.182	***	
SC1	<--	Social capital	0.917	1.089	0.016	66.766	***	
SC2	<--	Social capital	0.873	1.042	0.017	59.674	***	
SC3	<--	Social capital	0.868	0.986	0.017	59.007	***	
JN1	<--	Technological empowerment	0.926	1.000			***	
JN2	<--	Technological empowerment	0.747	0.762	0.016	46.945	***	
JN3	<--	Technological empowerment	0.919	1.022	0.013	76.465	***	
JN4	<--	Technological empowerment	0.919	0.985	0.013	76.657	***	
R1	<--	Pandemic resilience	0.898	1.000			***	
R2	<--	Pandemic resilience	0.878	1.084	0.017	63.687	***	
R3	<--	Pandemic resilience	0.878	0.998	0.016	63.777	***	
R4	<--	Pandemic resilience	0.881	1.109	0.017	64.289	***	
R5	<--	Pandemic resilience	0.904	1.092	0.016	68.617	***	
R6	<--	Pandemic resilience	0.914	1.114	0.016	70.760	***	
R7	<--	Pandemic resilience	0.885	1.010	0.016	64.916	***	
R8	<--	Pandemic resilience	0.905	1.079	0.016	68.897	***	
R9	<--	Pandemic resilience	0.918	1.062	0.015	71.475	***	
R10	<--	Pandemic resilience	0.923	1.048	0.014	72.511	***	
Social capital	<--	Technological empowerment	0.885	0.743	0.014	53.060	***	is
Pandemic resilience	<--	Social capital	0.667	0.691	0.021	33.301	***	is
Pandemic resilience	<--	Technological empowerment	0.325	0.283	0.016	18.047	***	is

SF, standardization factor; NSC, non-standardized coefficient; S.E., standard error; C.R., critical ratio (B/S.E.); ***, *p* < 0.001.

**Table 7:** Standardized direct, indirect, and total effects of the hypothesized model.

Path	Point Estimate	Product of Coefficients		Bootstrapping				Two-Tailed Significance
				Percentile 99% CI		Bias-Corrected Percentile 99% CI		
		SE	Z	Lower	Upper	Lower	Upper	
Standardized direct effects								
PR<--TE	0.325	0.029	11.207	0.272	0.386	0.271	0.385	0.002 (**)
Standardized indirect effects								
PR<--TE	0.590	0.025	23.6	0.538	0.638	0.539	0.639	0.002 (**)
Standardized total effects								
PR<--TE	0.915	0.007	130.714	0.901	0.929	0.899	0.929	0.002 (**)

Standardized estimating of 10,000 bootstrap samples; **, *p* < 0.01

**Table 8:** Results of hypotheses testing.

Hypothesis	Results
H1: Social capital → Pandemic Resilience (positive).	Supported
H2: Technological Empowerment → Pandemic Resilience (positive).	Supported
H3: Social capital plays an intermediary effect between technological empowerment and pandemic resilience.	Supported

## Data Availability

The data presented in this study are available on request from the corresponding author (tangjy21@m.fudan.edu.cn). The data are not publicly available due to privacy reasons.
